# Functional Investigation of the Tumoural Heterogeneity of Intrahepatic Cholangiocarcinoma by In Vivo PET-CT Navigation: A Proof-of-Concept Study

**DOI:** 10.3390/jcm11185451

**Published:** 2022-09-16

**Authors:** Luca Viganò, Egesta Lopci, Luca Di Tommaso, Annarita Destro, Alessio Aghemo, Lorenza Rimassa, Luigi Solbiati, Arturo Chiti, Guido Torzilli, Francesco Fiz

**Affiliations:** 1 Hepatobiliary Unit, Department of Minimally Invasive General & Oncologic Surgery, Humanitas Gavazzeni University Hospital, Viale M. Gavazzeni 21, 24125 Bergamo, Italy; 2 Department of Biomedical Sciences, Humanitas University, Viale Rita Levi Montalcini 4, 20090 Milan, Italy; 3 Department of Nuclear Medicine, Humanitas Research Hospital—IRCCS, 20089 Milan, Italy; 4 Pathology Unit, Humanitas Research Hospital—IRCCS, 20089 Milan, Italy; 5 Department of Internal Medicine, Division of Internal Medicine and Hepatology, Humanitas Research Hospital—IRCCS, 20089 Milan, Italy; 6 Medical Oncology and Hematology Unit, Humanitas Research Hospital—IRCCS, 20089 Milan, Italy; 7 Department of Radiology, Humanitas Research Hospital—IRCCS, 20089 Milan, Italy; 8 Department of Surgery, Division of Hepatobiliary and General Surgery, Humanitas Research Hospital—IRCCS, 20089 Milan, Italy; 9 Department of Nuclear Medicine and Clinical Molecular Imaging, University Hospital, 72076 Tübingen, Germany

**Keywords:** intra-tumoural heterogeneity, intrahepatic cholangiocarcinoma, positron emission tomography–computed tomography, immunology, FGFR2 translocation, imaging fusion, navigation technology

## Abstract

Intra-tumoural heterogeneity (IH) is a major determinant of resistance to therapy and outcomes but remains poorly translated into clinical practice. Intrahepatic cholangiocarcinoma (ICC) often presents as large heterogeneous masses at imaging. The present study proposed an innovative in vivo technique to functionally assess the IH of ICC. Preoperative 18F-FDG PET-CT and intraoperative ultrasonography were merged to perform the intraoperative navigation of functional tumour heterogeneity. The tumour areas with the highest and the lowest metabolism (SUV) at PET-CT were selected, identified during surgery, and sampled. Three consecutive patients underwent the procedure. The areas with the highest uptake at PET-CT had higher proliferation index (KI67) values and higher immune infiltration compared to areas with the lowest uptake. One of the patients showed a heterogeneous presence of FGFR2 translocation within the samples. Tumour heterogeneity at PET-CT may drive biopsy to sample the most informative ICC areas. Even more relevant, these preliminary data show the possibility of achieving a non-invasive evaluation of IH in ICC, paving the way for an imaging-based precision-medicine approach.

## 1. Introduction

Intra-tumoural heterogeneity (IH) is regarded as a major determinant of resistance to therapy and patients’ prognosis but remains poorly translated into clinical practice [1,2,3]. The evaluation of IH has been mostly determined from the laboratory analyses of resected specimens, while, more recently, much of the research has been concentrated on imaging. The possibility of achieving a non-invasive mapping of IH is extremely appealing, because it would allow a better characterisation of the tumour (IH-based biopsies), a more precise prediction of prognosis, and a more effective treatment (IH-based therapies). Progress in medical imaging modalities open new opportunities for the investigation of IH. Positron emission tomography–computed tomography (PET-CT) offers unique functional imaging of liver tumours [4,5,6]. Navigation technology systems [7,8] may merge different imaging modalities (morphologic and functional ones) to optimise the identification of the different tumour areas. 

Among liver tumours, intrahepatic cholangiocellular carcinoma (ICC) is probably the most adequate to study IH. It is often diagnosed at an advanced stage and presents as large heterogeneous masses with a non-homogeneous uptake at PET-CT [9]. This presentation at imaging corresponds to a major genetic IH [10,11], even if the two have never been associated. 

The present study depicts an innovative in vivo technique to functionally study the IH of ICC. Preoperative 18F-fluorodeoxyglucose (FDG) PET-CT images were merged with ultrasonography ones to navigate the tumour and to precisely explore the association between IH at imaging and IH at pathology. In this proof-of-concept study, we tested the procedure during surgery to unequivocally evaluate its reliability and accuracy.

## 2. Materials and Methods

All consecutive patients affected by ICC and undergoing surgery were considered. Inclusion criteria were (a) aged ≥18 years; (b) ICC size >50 mm; (c) preoperative PET-CT with evidence of tumour areas having a heterogeneous uptake. Exclusion criteria were: (a) diagnosis of mixed hepato-cholangiocarcinoma at the final pathology; (b) preoperative chemotherapy or any preoperative loco-regional treatment, including thermal ablation, chemoembolisation, or radioembolisation; (c) uncontrolled diabetes or any metabolic alteration preventing an accurate SUV evaluation. The standard preoperative imaging included thoracoabdominal CT and hepatic magnetic resonance imaging (MRI). A multidisciplinary team discussed the management of every patient. Informed consent was signed by all the participants. The local ethics committee approved the study (approval number: 146/20 on 20 February 2020). 

### 2.1. PET-CT Imaging

FDG PET-CT were performed on General Electric Discovery 690 (General Electric Healthcare, Waukesha, WI, USA) according to standard procedures. Reconstructed images were examined and interpreted by an experienced nuclear medicine physician (EL). The tumour areas with different metabolic activities at PET-CT were preoperatively identified. In each ICC, we considered sampling only the spots with the highest and with the lowest standardised uptake value (SUVmax and SUVmin, respectively) within the tumour. The low-uptake areas corresponding to necrosis at morphologic imaging (CT and MRI) were not considered.

### 2.2. IOUS and Intraoperative Navigation

At the point of laparotomy, intraoperative ultrasonography (IOUS) was performed using an Esaote Twice ultrasound system (Esaote, Genoa, Italy), equipped with an intraoperative T-shaped probe (IOT332 probe, Esaote, Genoa, Italy), working at 3–11 MHz frequency.

**Figure 1:**
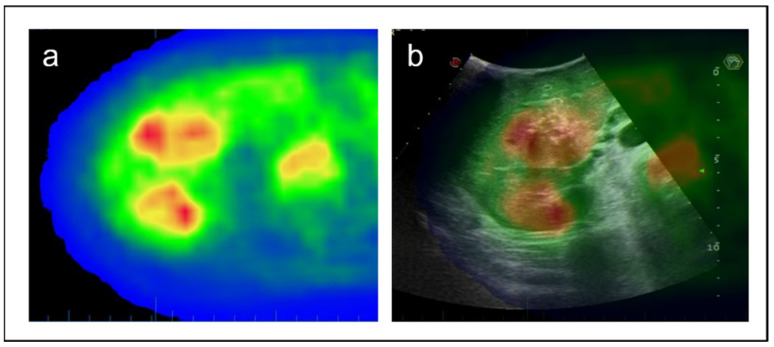
Navigation technology with the intraoperative fusion of preoperative PET-CT and IOUS. The tumour areas having different uptake at PET-CT are identified in vivo during surgery. (**a**) PET axial view of ICC; (**b**) Intraoperative fusion of PET-CT and IOUS images.

**Figure 2:**
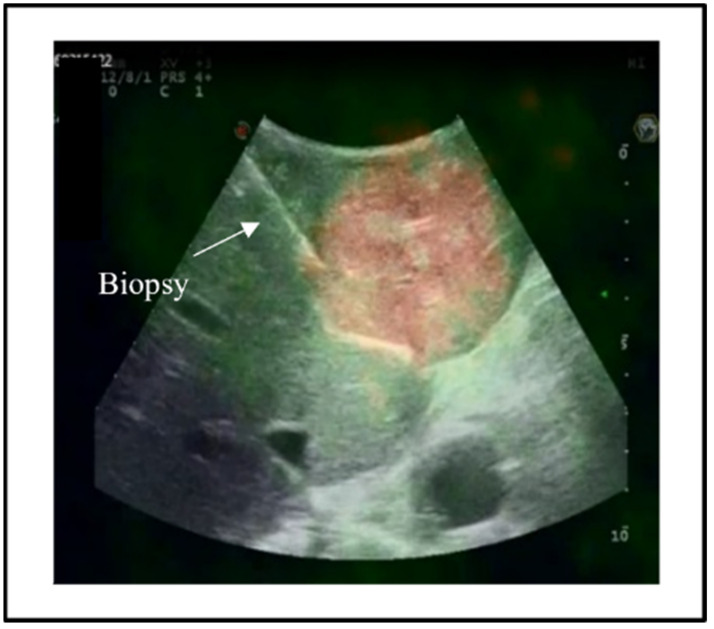
IOUS-guided biopsy of the tumour areas having different uptake at PET-CT.

The two imaging modalities (PET-CT and IOUS) were synchronised by a semi-automatic system in the following steps. The images of PET-CT were uploaded to the ultrasound machine and then projected onto the screen beside the standard IOUS images. Some intrahepatic anatomic landmarks identified on the PET-CT (e.g., umbilical portion, first-order bifurcation of the right portal branch, hepato-caval confluence) were manually identified at IOUS. Once a landmark was visualised at IOUS, the axial image of PET-CT with the same landmark was identified and selected, and a mark was placed on the anatomical structure in the two imaging modalities. After the identification and selection of two landmarks, the machine provided an automatic synchronisation of the two imaging modalities. The correct synchronisation between the two imaging modalities was then verified by scanning all the liver. If any discordance was observed, some additional anatomic landmarks were selected to refine the process until a perfect overlapping of all intrahepatic anatomical structures was achieved. Once the process was completed, the overlapping of the full liver was obtained, and the PET-CT navigation was possible (Figure 1). The SUVmax and the SUVmin tumour areas selected at PET-CT were identified and sampled using a 16-gauge Trucut needle (Figure 2); the needle trajectory was selected to be fully included within the resected portion of the liver to avoid any risk of tumour-seeding in the future liver remnant. At least two biopsies were taken from each area. At the end of the resection, the same targeted areas were again identified, and a macrobiopsy was performed. Only samples with an adequate cellular composition were retained for the analyses (through a quick histologic check after sampling). 

### 2.3. Pathology Analyses

The specimens were fixed in formalin, paraffin-embedded, and stained with haematoxylin-eosin. Each sample had a standard morphological evaluation. Immunohistochemistry (IHC) was used to analyse the following parameters: the expression of CK7 and CK19; immune infiltrate (CD3+, T-lymphocyte marker; CD4+, helper/inducer T-lymphocyte marker; CD8+, suppressor/cytotoxic T-lymphocyte marker; CD68+, macrophage marker; and CD163+, M2 macrophage marker); the expression of programmed cell death protein 1 (PD1), its ligand (PD-L1), and tumour protein p53; proliferation index (Ki67); and metabolic enzymes glucose-6-phosphate dehydrogenase (G6PD) and citrate synthase (CS). We analysed the presence of FGFR2 translocations and the presence of microsatellite instability, and the loss of heterozygosity (1p36) using fluorescence in situ hybridisation. For PD1, PD-L1, p53, Ki67, G6PD, and CS, data were expressed as the percentage of immunoreactive cells compared to the total number of neoplastic cells. For the immune infiltrate (CD3, CD4, CD8, CD68, CD163), data were expressed as the percentage of immunoreactive cells compared to the total number of immune cells.

The specimens from SUVmax and SUVmin areas were separately analysed, and their data were compared. Both IOUS-guided tumour biopsies before resection and macroscopical biopsies at the end of resection were analysed. The concordance between samples from the same area was assessed.

## 3. Results

We enrolled three consecutive patients with a diagnosis of ICC confirmed at pathology. Table 1 summarises the patients’ characteristics. The mean tumour size was 92 mm (range 60–120). At PET-CT, the mean SUVmax was 11.2 (8.9–14.7), the mean SUVmin was 5.3 (5.1–5.5), and the mean difference between the two was 5.8 (3.5–9.6). The synchronisation of PET-CT with IOUS and its navigation was successful in all patients. 

Table 2 summarises the pathology data. The IH of ICC was evident in different analyses. One patient had a lower tumour grading in the SUVmin area than in the SUVmax one (G1 vs. G2). One patient had a phenotypic IH, i.e., variable CK19 positivity in areas with a different uptake. One patient had a molecular IH: FGFR2 translocation was evident in the high-uptake area, while it was not in the low-uptake one. PET-CT uptake was also associated with the proliferative index in two patients (70% in the SUVmax area vs. 10% in the SUVmin area of one patient; 70 vs. 20%, respectively, in one). Finally, IH on PET-CT corresponded to heterogeneous immune infiltration: SUVmax areas had a higher CD8+ infiltrate in all patients (a mean of 15 vs. 8%), and a higher CD4+ (30 vs. 10%), CD68+ (25 vs. 10%), and CD163+ (30 vs. 12%) infiltrate in two patients. Metabolic indexes, PD1, PD-L1, and p53 expression were similar between areas. 

The pathology data of IOUS-guided biopsies and macrobiopsies after resection obtained from the same area were concordant.

## 4. Discussion

ICC is an aggressive malignancy with a poor prognosis. Standard chemotherapy has scarce disease control [12], but targeted therapies and immunotherapy could change this scenario. Some of the commonest ICC mutations concern the p53 pathway, Ras/Raf/MEK/ERK pathway, metabolic pathway (IDH1/IDH2), FGFR2, and 1p36 [10,11,13]. To date, targeted therapies for FGFR2 rearrangements and IDH1 mutations have been approved, and some other drugs have had tissue-agnostic approval [14,15]. However, the effectiveness of systemic therapies is limited by profound tumour genetic heterogeneity [10,11]. Walter et al. depicted varying expression patterns of MSH6 (mismatch repair protein) in peripheral and central areas of ICC [16]. Goyal et al. reported intra-tumoural clonal heterogeneity, in terms of acquired resistance to FGFR inhibition, in patients with FGFR2-fusion-positive tumours [17]. The possibility of predicting IH with non-invasive imaging is of major interest but has not been demonstrated yet.

ICC malignant cells have increased their expression of glucose transporters and a high activity of hexokinase, which leads to augmented glucose metabolism [4]. It corresponds to an increased FDG accumulation at PET-CT, especially in moderately and poorly differentiated ICCs [5]. High glucose metabolism in ICC is expected to be associated with increased tumour aggressiveness. Indeed, Seo et al. reported a high SUV as an independent predictor of postoperative recurrence [6].

We investigated the association between the heterogeneous uptake of ICC at PET-CT and IH. Among liver tumours, ICC is the most adequate for this analysis: it is usually diagnosed at an advanced stage (large masses); FDG PET-CT uptake is often non-homogeneous; and resectable patients do not receive preoperative chemotherapy, which could compromise PET-CT findings. Navigation technology provided a fundamental contribution. It is commonly used to guide the percutaneous interstitial treatment of tumours not visible on ultrasound [7]. In liver surgery, navigation technology merges preoperative and intraoperative imaging to identify the anatomy and the correct plane [8]. We used the fusion of preoperative PET-CT with IOUS to have an accurate identification of tumour areas with a different uptake at PET-CT. The intraoperative analysis allowed us to maximise the precision of the biopsy and unequivocally ascertain the capability of PET-driven biopsies to detect IH. 

In the present series, PET-CT effectively caught IH. FDG uptake was associated with proliferative index (Ki67) and tumour grading: the areas with the highest SUV were the most aggressive parts of ICC. We also observed interesting results concerning genetic mutations and immune infiltration. In one patient, PET-CT identified a heterogeneous mutational status of FGFR2 (a wild-type in the SUVmin area and translocated in the SUVmax one). The remaining two patients had a wild-type status of FGFR2 in all biopsies. Considering immune infiltration, tumour areas with a higher FDG uptake had higher levels of T-lymphocytes (CD3+ and CD4+/CD8+) and macrophages (CD68+/CD163+) compared to areas with a lower uptake. Those data are clinically relevant: FGFR2 mutations are the target of approved drugs [14,18,19], and the immune infiltrate is a major determinant of prognosis in ICC patients [20,21]. A further result deserves consideration. In general, the key enzymes of anaerobic glycolysis and mitochondrial respiration (G6PD and CS, respectively) did not show a clear association with the SUV. Even if FDG PET-CT detects an augmented glucose metabolism [22], the different uptake did not always correspond to a heterogeneous metabolic pattern. Due to the study design (three patients), we can formulate some hypotheses about the mechanisms underlying the heterogeneous FDG uptake, also considering that they can vary among patients. In one patient, the expression of citrate synthase, which is used in oxygen-dependent ATP production, dropped in the high-SUV area. This finding is consistent with the concept of increased glucose consumption in the hypoxic areas of the tumours, which are forced to switch to the energy-inefficient anaerobic glycolysis and thus require many times more substrate for the same ATP output [23]. In the remaining two patients, the high-uptake areas were probably related to a major increase in the proliferative index (of both patients) and an FGFR2 translocation (in one). Investigations on the link between the latter gene and glucose metabolism are thus far limited, but the FGF/FGFR pathway involves anti-apoptosis signalling, proliferation, and angiogenesis [24]. 

The present study is in line with modern oncological research. Advanced imaging and analyses achieved excellent results for ICC, being able to provide a non-invasive prediction of tumour pathology data and prognosis [25,26]. Focusing on PET-CT, Yugawa et al. demonstrated that FDG uptake is associated with immune infiltration [27]. Fiz et al. reported that the radiomic analysis of the ICC and peritumoral tissue accurately predicts tumour grading, microvascular invasion, and survival [28]. Our preliminary data are coherent with such literature but represent a major step forward, thanks to IH mapping. 

The proposed approach is clinically relevant for at least two reasons. First, the fusion of two different imaging modalities—morphological (ultrasound) and functional (PET)—provided a non-invasive depiction of ICC heterogeneity and detection of the most significant tumour portions. Even if our data are preliminary, the concordance of these results among multiple samplings from the same area strengthens the reliability and the reproducibility of the present technique. PET-driven biopsies could become a new standard in ICC patients: to catch the most relevant and aggressive areas of the tumour, have a more precise prediction of prognosis, and schedule a more effective patient-tailored treatment. Theoretically, the same approach could be applied to other liver tumours (primary or metastatic) and tracers. Liver metastases from colorectal cancers have both an intense FDG uptake with heterogeneous areas and a proven intralesional heterogeneity that correlates with prognosis [29,30,31]. Metastatic neuroendocrine tumours have a known inter-lesional heterogeneous DOTA peptides uptake, which can bear relevance for treatment strategies [32]. The tracers of the PSMA molecules can visualise the heterogeneity of prostate cancer metastases and, more recently, primary hepatic malignancies [33,34]. The uptake can depict variations in vascularity across the tumoural volume [34].

Second, the present technique was the first one that reliably associates the different FDG-uptake areas with tumour heterogeneity at a phenotypic, molecular, and genetic level and with immune infiltration. Our experience only provides a preliminary exploration of the concept but could be the basis for a better understanding of IH, a precision-medicine approach, and the identification of new biomarkers and therapeutic targets. By analysing a larger population, we could identify the SUV values and patterns which are able to non-invasively predict tumour characteristics. 

## 5. Conclusions

The present study demonstrates that the fusion of morphological and functional imaging modalities may allow an in vivo and reliable evaluation of tumour heterogeneity. Discrepant intra-tumoural phenotypic, molecular, and genetic patterns were identified, as well as heterogeneous immune infiltrations. The proposed approach could increase the efficacy of percutaneous biopsies and could be the basis for a better understanding of IH. 

## Figures and Tables

**Table 1:** Clinical characteristics of the patients.

	Patient #1	Patient #2	Patient #3
Age	76	60	71
Sex	Male	Male	Male
Tumour size, mm	120	60	97
Number of tumours	1	1	1
Grading	G2	G2	G3
Surgical margin, mm	3	10	1
Microscopic vascular invasion	Y	N	Y
Perineural infiltration	N	N	Y

**Table 2:** Summary of the pathology results.

	Patient #1		Patient #2		Patient #3	
	Area SUV Min	Area SUV Max	Area SUV Min	Area SUV Max	Area SUV Min	Area SUV Max
**SUV**	5.1	14.7	5.5	9.9	5.4	8.9
**Morphology**	stroma < cells	cells > stroma	cells = stroma	cells = stroma	cells = stroma	cells = stroma
**Phenotype**	CK7^+^ CK19^−/+^	CK7^+^ CK19^−/+^	CK7^+^ CK19^/+^	CK7^+^ CK19^−/+^	CK7^+^ CK19^−/+^	CK7^+^ CK19^−^
**Grading**	G1	G2	G2	G2	G3	G3
**Proliferation index (KI67)**	15%	15%	10%	70%	20%	70%
**P53**	20%	10%	30%	30%	60%	60%
**PDL-1**	Neg	Neg	Neg	Neg	Neg	Neg
**PD1**	5%	Neg	5%	10%	5%	Neg
**FGFR2**	WT	WT	WT	Translocated	WT	WT
**1p36**	LOH	LOH	Conserved	Conserved	Conserved	Conserved
**Immune infiltrate**						
**CD3**	10%	10%	10%	10%	10%	20%
**CD4**	10%	20%	10%	40%	20%	20%
**CD8**	5%	10%	10%	20%	5%	15%
**CD68**	10%	20%	10%	30%	20%	20%
**CD163**	20%	20%	20%	50%	5%	10%
**Metabolic indexes**						
**G6PD**	80%	80%	40%	100%	50%	50%
**CS**	100%	60%	80%	100%	20%	20%

**CD163**, marker of M2 macrophages; **CD3** (cluster of differentiation 3), marker of T-lymphocytes; **CD4**, marker of helper/inducer T-lymphocyte; **CD68**, pan-macrophage or M1 marker; **CD8**, marker of suppressor/cytotoxic T-lymphocyte; **CK19**, cytokeratin 19; **CK7**, cytokeratin 7; **CS**, citrate synthase; **FGFR2**, fibroblast growth factor receptor 2; **G6PD**, glucose-6-phosphate dehydrogenase; **Ki67**, proliferation index; **LOH**, loss of heterozygosity; **Neg**, negative; **p53**, tumour suppressor protein; **PD-1**, programmed cell death protein 1; **PD-L1**, programmed death ligand 1; **SUV**, (standardised uptake value) semiquantitative parameter of FDG uptake; **WT**, wild type.

## Data Availability

The data presented in this study are available from the corresponding author on reasonable request.
